# Dynamic resectorization to improve utility of healthcare systems

**DOI:** 10.1186/s41043-024-00594-4

**Published:** 2024-07-05

**Authors:** Aydin Teymourifar, Maria A. M. Trindade

**Affiliations:** 1 Universidade Católica Portuguesa, Católica Porto Business School, Centro de Estudos em Gestão e Economia, Porto, Portugal; 2 SDA Bocconi, School of Management, Milano, Italy

**Keywords:** Healthcare system, Resectorization, Hospitals’ Closure, Balancing, Utility, Loss of demand, Simulation

## Abstract

Balancing is an essential challenge in healthcare systems that requires effective strategies. This study aims to address this crucial issue by suggesting a practical approach. We show the potential of balancing a regional healthcare system to improve its utility. We consider a regional healthcare system comprising multiple hospitals with different sizes, capacities, quality of service, and accessibility. We define a utility function for the system based on the sectorization concept, which endeavors to form a balance between hospitals in terms of essential outputs such as waiting times and demands. The dynamic nature of the system means that this balance degrades over time, necessitating periodic sectorization, which is called resectorization. Our methodology stands out for incorporating resectorization as a dynamic strategy, enabling more flexible and responsive adaptations to continuously changing healthcare needs. Unlike previous studies, based on a system-oriented approach, our resectorization scenarios include the periodic closure of some hospitals. This enables us to enhance both the capacity and quality of healthcare facilities. Furthermore, in contrast to other studies, we investigate the states of diminishing demand throughout the resectorization process. To provide empirical insights, we conduct a simulation using data from a real-world case study. Our analysis spans multiple time periods, enabling us to dynamically quantify the utility of the healthcare system. The numerical findings demonstrate that substantial utility improvements are attainable through the defined scenarios. The study suggests a practical solution to the critical challenge of balancing issues in regional healthcare systems.

## Introduction

In healthcare systems, besides less average waiting time and higher average quality [28], balancing between service centers in terms of quality and demand is desired. The balancing should be implemented based on policies that benefit patients, taking into consideration their preferences and needs [31, 32]; otherwise, it cannot be valuable and applicable [42]. Our study is developed around the concept of sectorization. The primary aim of sectorization is commonly defined as dividing a large region into smaller and balancing sectors; however, it encompasses a broader range of implications that vary depending on the context [44]. It has several applications in political districting [25], supply chain network design [48], elderly care service districting [24], emergency medical service districting [34], power distribution networks [6], police districting [23], water distribution network design [46], commercial districting [29], and school districting [11]. As demonstrated by these examples, sectorization is closely related to the concept of districting, but it also intersects with balancing [7–9]. Sectors typically are desired to be balanced based on different criteria. In sectorization terminology, this is often referred to as achieving equilibrium among sectors [44]. In our study, each hospital and its corresponding patient group are treated as a sector, with a particular emphasis on balancing the demand and waiting times between these sectors. We underline that in this study, the term ’sectorization’ is not used in its geographical sense. Instead, it is used to characterize balancing between sectors [42]. Balancing can also ensure that services within sectors are equitably accessible [3].

This work discusses a regional healthcare system that contains hospitals with different features. Since service systems are generally dynamic, the desired condition may be lost over time, so that resectorization can reform it [42]. Resectorization is not a concept different from sectorization; it is just for balance over time. We define a utility function for the discussed system based on a multi-objective (MO) model [26]. We employ the simulation method to solve the model [43]. The sectorization models designed based on integer programming techniques are non-convex. In this case, it can be challenging to find solutions for them (Teymourifar, 2021). Simulation models do not have this deficiency. Furthermore, simulation can be a practical tool for solving dynamic sectorization models [42] in healthcare management [2, 10, 12, 14, 45]. In the dynamic model of this study, scenarios such as closing a subset of hospitals and growing capacity and quality levels are analyzed to improve utility.

The outlines of this work, its similarities and distinctions from earlier works can be summarized as follows:

- We determine a function for patients based on which they choose a hospital. Some studies in the literature have similar definitions [16, 17, 38]. Unlike them, we don’t include pricing decisions in the model but consider the decision of using or closing the hospitals over different periods. This kind of facility layout decision has been investigated deeply in the literature on healthcare management [13, 36]. However, different from many of the works in this area, we regard the quality of hospitals and the distance of patients to them for the decisions. We indicate that closing a subset of hospitals can be beneficial in terms of providing a more balanced system.
- We define the utility of a healthcare system based on sectorization. Earlier studies specified utility for individuals and/or society, not for the healthcare system [16, 17, 38, 42].
- Unlike previous studies, we aim to balance a healthcare system in terms of both demands and waiting times. Advancing average quality is also desired.
- We consider the number of patients who exit the system, a matter that has been neglected in most previous works.
- Unlike the former works, we do resectorization for more than one period. This point makes the model more suitable for real-life applications.
- Unlike the former works, we investigate the effects of hospital closures over various periods.

Although the closure of hospitals has not been investigated enough in the literature, it holds significance for practical reasons. In healthcare planning and management, strategic decisions about resource allocation are paramount. When a hospital is closed for a period of time, its resources, including staff, equipment, and services, can be redistributed to other facilities. Dynamic capacity sharing [21] can be beneficial, mainly if the resources are used to flexibly meet demand in other parts of the system. Considering the periodic closure of some hospitals allows us to examine scenarios where resources could be reallocated more efficiently. It should be noted that periodically closing hospitals is not actually a form of dynamic capacity sharing, but reallocating resources following the closure may benefit the healthcare system as a whole.

Other parts of the paper are organized as follows: Section 2 presents a literature review on the topic. Section 3 describes the proposed model. The case study, scenarios, and obtained results are presented in Section 4. The explanation of conclusions and future works in Section 5 constitutes the last part of the study.

## Literature review

Efficient, equitable, and quality-focused access to hospitals represents a critical challenge faced by societies globally [5]. A challenging subject in healthcare systems is providing a balance between hospitals in the system. The concept of sectorization, also known as districting [18, 19], can be applied in developing models to achieve balance in healthcare systems. Sectorization involves the initial allocation and organization of resources, sorting them into different sectors based on medical specialties or service areas [35]. In this study, we use the term “resectorization” which deals with the ongoing process of reevaluating and potentially adjusting resource allocation and organization in response to changing circumstances [35]. This adaptability is crucial as the state of healthcare systems changes over time. Resectorization helps healthcare systems adapt to changes and align services with current healthcare demands. Resectorization represents a form of sectorization dynamically applied over time [42].

In the literature on healthcare management, several dynamic models have been presented to enhance the outputs of healthcare systems, especially patient care [33]. Kwok et al. [20] introduce a dynamic model to evaluate patient care and resource utilization. It enhances the healthcare system by considering customized evaluation criteria. Huirne et al. [15] use dynamic programming to optimize healthcare decisions, focusing on cost reduction, improved quality of life, and patient survival. Carbone et al. [4] emphasize adapting treatments based on prior interventions, enhancing treatment efficacy, and reducing adverse effects. Nguyen et al. [27] employ recurrent neural networks to predict health trajectories, offering promise for predictive tasks such as risk prediction and readmission forecasting. These studies represent the evolution of healthcare systems’ dynamic modeling to improve patient care.

Recent studies in the literature deal with this subject from different perspectives. An article by Taymaz et al. [36] presents a stochastic optimization model for locating walk-in clinics in a network to cater to mobile populations. The authors model the continuum of care required for different diseases using coverage definitions that reflect the adherence protocols for the services [36]. The objective is to maximize the total expected weighted coverage of the network, subject to a conditional value-at-risk measure [36]. The article develops coverage definitions and an optimization model and presents a computational study carried out on a real-life case in Africa [36].

A study by Kaya et al. [16] investigates the coexistence of public and private hospitals and proposes subsidy mechanisms to balance their capacity utilization and improve overall access to healthcare. The authors develop a simulation model and analyze the effects of different public policies on patients’ preferences, social utility, public healthcare spending, patient satisfaction, and waiting times [16]. The study sheds light on the importance of balancing public and private hospitals to improve access to healthcare and patient satisfaction. It provides insights into the impact of different policies on the healthcare system [16].

Similarly, a study by Kaya et al. [17] proposes new contract mechanisms based on pricing and subsidy policies to balance healthcare systems containing public and private hospitals. The study formulates a multi-objective problem and proposes analytical models to determine the best contract mechanisms and optimal contract parameters to maximize total social utility [17]. The study presents detailed numerical results and highlights that the proposed mechanisms can significantly improve system performance, reduce waiting times, and increase patient satisfaction.

The study by Teymourifar et al. [41] addresses the challenge of balancing healthcare systems comprising both public and private hospitals. The study underscores the existing preference of patients towards public hospitals, resulting in overcrowding and dissatisfaction, despite the better services offered by private hospitals with shorter waiting times, albeit at higher prices [41]. The study proposes new pricing policies and contract mechanisms between the government and private hospitals to alleviate the situation [41]. An MO problem is formulated, and analytical models are presented to optimize the contract mechanisms and parameters to maximize the total social utility [41]. The study provides a detailed numerical analysis to compare the effectiveness of the different contract mechanisms. It highlights the significant improvements in system performance, waiting times, and patient satisfaction with the proposed mechanisms [41].

In a recent study, Teymourifar [42] proposes a new model for balancing the accessibility of healthcare services in a region based on resectorization. The study focuses on dividing a vast region into symmetric sectors and balancing the accessibility of healthcare units, which is a common goal of governments. The proposed model considers each hospital and the patients that choose it as a sector, and it defines a new bi-objective function that aims to improve quality and accessibility. Simulation-based optimization is used for resectorization, portrayed as a tool for policymaking based on contract mechanisms. The experimental results showed that it is achievable to balance the accessibility of healthcare units with a suitable contract mechanism.

The topic of hospital closures has not been extensively explored in the healthcare management literature. However, in one of the notable exceptions, Saghafian et al. [30] discuss the significant number of hospital closures in the United States, a trend that has accelerated during the COVID-19 pandemic. This trend has been driven by financial hardships hospitals face, including reduced patient volume and elective surgery cases, as well as the financial challenges of treating COVID-19 patients. The paper addresses this trend’s concerns for patients, healthcare providers, and policymakers. It highlights the need for policymakers to be informed by research on the implications, trade-offs, and consequences of various strategies related to hospital closures. As previously discussed, while hospital closure over periods is not synonymous with dynamic capacity sharing [21], it can indeed act as a catalyst for this process. The redistribution and reallocation of resources following a hospital closure can enhance the dynamic sharing of capacity across the remaining healthcare infrastructure.

As seen in Table 1, this work offers new insights into healthcare management and presents a more comprehensive framework for system balancing. It presents a novel approach to balancing a healthcare system by considering various factors. Specifically, we develop a function that captures patients’ hospital preferences based on quality and distance, and we explore the impact of closing certain hospitals on system balance. Unlike some related studies, we do not include pricing decisions in our model but focus on facility layout decisions and their effects on demands, waiting times, and patient exits. Additionally, we define the utility of the healthcare system itself, rather than just individuals or society, and we sectorize for multiple periods to better reflect real-life applications. This emphasis aims to contribute to a more nuanced understanding of the healthcare system by examining these aspects.

As seen in Table 1, in the existing literature, the work most closely related to our inquiry is that of Teymourifar [42]. However, the distinctions between the current study and the mentioned one are noteworthy, which can be articulated as follows: This work conceptualizes a utility function specifically tailored for the healthcare system rather than individual patients or society. Furthermore, this study extends the scope of analysis by conducting resectorization over several time periods. It also incorporates decisions of hospital closure alongside considering the loss of demand, providing a comprehensive view of the system’s dynamics.

**Table 1:** A comparison between studies in the literature and our work

	Kaya et al. (2020a)	Saghafian et al. (2022)	Taymaz et al. (2020)	Teymourifar et al. (2020)	Teymourifar (2022a)	This paper
Patients oriented					$\surd$	
System oriented	$\surd$		$\surd$	$\surd$		$\surd$
Patients’ preferences	$\surd$			$\surd$	$\surd$	$\surd$
Pricing	$\surd$			$\surd$	$\surd$	
Public expenditures	$\surd$	$\surd$		$\surd$	$\surd$	
Patient satisfaction	$\surd$			$\surd$	$\surd$	
Waiting times	$\surd$	$\surd$		$\surd$	$\surd$	$\surd$
Hospital distance		$\surd$			$\surd$	$\surd$
Quality of service	$\surd$	$\surd$		$\surd$	$\surd$	$\surd$
Utility function	$\surd$			$\surd$	$\surd$	$\surd$
Sectorization					$\surd$	$\surd$
Multiple hospitals	$\surd$	$\surd$	$\surd$	$\surd$	$\surd$	$\surd$
Multiple periods						$\surd$
Loss of demand		$\surd$				$\surd$
Hospital closure		$\surd$				$\surd$

## Description of the model

**Fig. 1:**
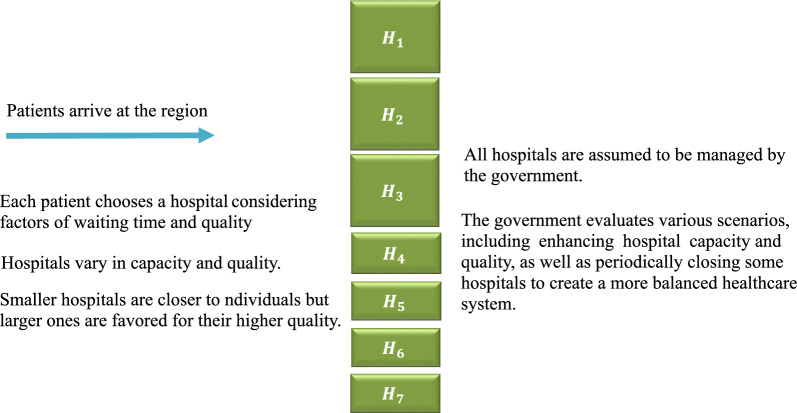
The overview of the model

As seen in Fig. 1, the problem of this study can be summarized as follows: A regional healthcare system is considered, which is comprised of hospitals with different characteristics. Some hospitals are small and have low capacity and quality, while others are large and have high capacity and quality. Even though individuals have a shorter distance to smaller hospitals, larger hospitals are preferred since they are better in terms of quality. All hospitals in the model are supposed to be administered by the government, which examines scenarios involving improving hospital capacity and quality as well as periodically closing some hospitals to provide a more balanced system.

Sectorization needs to be dynamically managed to adapt to changing conditions over time, which is also known as resectorization. As mentioned before, it’s important to note that in this context, sectorization does not refer to geographical aspects but rather to balancing sectors in terms of demand and waiting time. Each sector is a hospital and the group of patients who choose it. As seen in Fig. 1, the government monitors patients’ choices and conducts evaluations across different scenarios, such as enhancing hospital capacity and quality and periodically closing some hospitals.

It’s important to emphasize that most examples from the literature cited in the first paragraph of the Introduction section do not reflect customers’ or patients’ preferences. As seen in Fig. 1, this study defines sectorization based on patients’ choices rather than mere allocation.

The following parts of this section elaborate on the proposed model. The used notations are summarized in Table 2.

**Table 2:** Used notations

Notation	Description	Type
$SI^t$	Set of patients in period *t*	Set
*SJ*	Set of hospitals	Set
$SO^t$	Set of open hospitals in period *t*	Set
*ST*	Set of periods	Set
*i*	Index of patients	Index
*j* and *k*	indexes of hospitals	Index
*J*	Number of hospitals	Parameter
$H_j$	Hospital *j*	Notation
*t*	Notation of period	Notation
*T*	Number of periods	Parameter
$y^t_j$	Decision variable about opening $H_j$ in period *t*	Decision variable
$x^t_{ij}$	Decision variable about selecting $H_j$ by the *i*-th patient in period *t*	Decision variable
$I^t_j$	Number of patients selecting $H_j$ in period *t*	Variable
$I^t$	Number of patients in period *t*	Variable
*I*	Number of patients in all periods	Variable
$p^t_j$	Probability of selecting $H_j$ in period *t*	Variable
${\bar{p}}^t$	Average probability of selecting hospitals in period *t*	Variable
$I^t_{out}$	Number of patients that the system loses in period *t*	Variable
$q^t_j$	Service quality level in $H_j$ in period *t*	Parameter
$TQ_j^t$	Total quality received by patients in $H_j$ in period *t*	Variable
$TQ^t$	Total quality received by patients in period *t*	Variable
$m^t_j$	Capacity of $H_j$ in period *t*	Parameter
$w^t_{ij}$	Waiting time of the *i*-th patient in $H_j$ in period *t*	Variable
${\bar{w}}^t_j$	Average waiting time in $H_j$ in period *t*	Variable
$wd^t_{ij}$	Time to reach $H_j$ by the *i*-th patient in period *t*	Variable
${\overline{wd}}^t_j$	Average time to reach $H_j$ in period *t*	Variable
${\overline{TW}}_j^t$	Average total time before examination in $H_j$	Variable
	(including reaching hospital and waiting for examination) in period *t*	
${\overline{TW}}^t$	Average total time before examination in all hospitals	Variable
	(including reaching hospital and waiting for examination) in period *t*	
$sn^t_{qu_i}$	Quality sensitivity of the *i*-th patient in period *t*	Parameter
$sn^t_{wt_i}$	Waiting time sensitivity of the *i*-th patient in period *t*	Parameter
$c_j$	Fixed costs of $H_j$	Parameter
$c^{ca}_j$	Cost of unit capacity in $H_j$	Parameter
$cq_j^{t}$	Cost of increasing quality in $H_j$ in period *t*	Variable
$cq^{t}$	Cost of increasing quality in all hospitals in period *t*	Variable
$c^t_{max}$	Upper level of the total cost in period *t*	Parameter
$U^t$	Utility function in period *t*	Objective function
*o*	Index of component *o* of $U^t$	Index
$f_o^t$	Component *o* of $U^t$	Objective function
*U*	Utility function for all periods	Objective function
$f_o$	Component *o* of *U*	Objective function
$S^t_s$	Scenario *s* in period *t*	Notation

We assume there are hospitals in a district, all managed by the government. The set of hospitals and the *j*-th hospital are shown as *SJ* and $H_j$, respectively. There are differences between hospitals in capacity, quality, and the average distance of patients to them. In period *t*, the quality and capacity of $H_j$ are demonstrated as $q^t_j$ and $m^t_j$, respectively.

In some periods, a subset of the hospitals may be closed. In period *t*, the decision variable about whether hospitals are open is expressed as in Equation 1.

1 $$\begin{aligned} y^t_j={\left\{ \begin{array}{ll} 1, &{} \text{ if } \hspace{0.1cm} H_j \hspace{0.1cm} is \hspace{0.1cm} open \hspace{0.1cm} in \hspace{0.1cm} period \hspace{0.1cm} \textit{t}\\ 0, &{} \text{ otherwise }. \end{array}\right. } \hspace{0.4cm} \forall j \in SJ, \forall t \in ST. \end{aligned}$$

$SO^t$ denotes the set of open hospitals in period *t*. It is obvious that $j \in SO^t$ if $y^t_j=1$, $\forall t \in ST$. The sensitivity of patient *i* to distance and quality in period *t*, her distance from $H_j$ and the related waiting time are respectively indicated as $sn^t_{wt_i}$, $sn^t_{qu_i}$, $wd^t_{ij}$ and $w^t_{ij}$ [42]. Patient *i* selects $H_j$ in period *t* if Inequality 2 is valid.

2 $$\begin{aligned} \begin{aligned} sn^t_{qu_i}q^t_j-sn^t_{wt_i}(w^t_{ij}+wd^t_{ij}) \ge \\ sn^t_{qu_{i}}q^t_k-sn^t_{wt_{i}}(w^t_{ik}+wd^t_{ik})\\ \hspace{0.01cm} \forall i \in SI, \hspace{0.1cm} \forall j \ne k \in SO^t \\ \end{aligned} \end{aligned}$$

Inequality 2 establishes the basis for patients’ preferences within our model, according to which patients choose their hospital. A patient leaves the system when Inequality 2 is not valid for any *j*. This situation can be interpreted as preferring another hospital outside of the region. The values of the decision variable defined in Equation 3 are determined by Inequality 2.

3 $$\begin{aligned} x^t_{ij}={\left\{ \begin{array}{ll} 1, &{} \text{ if } \hspace{0.1cm} patient \hspace{0.1cm} i \hspace{0.1cm} selects \hspace{0.1cm} H_j \hspace{0.1cm} in \hspace{0.1cm} period \hspace{0.1cm} \textit{t}\\ 0, &{} \text{ otherwise }. \end{array}\right. } \forall i \in SI^t, \forall j \in SO^t, \forall t \in ST. \end{aligned}$$

It should be noted that the “decision variable” specifically denotes elements that are related to a decision-making process, such as a government’s decision to close a hospital or a patient’s choice of which hospital to attend. However, “variables” generally refer to the outputs produced by the simulation process.

The number of patients that prefer $H_j$ in period *t* is acquired as in Equation 4.

4 $$\begin{aligned} I^t_j = \sum _{i \in SI^t} x^t_{ij}, \hspace{0.15cm} \forall j \in SO^t, \forall t \in ST. \end{aligned}$$

The probability of selecting $H_j$ in period *t* is defined as in Equation 5.

5 $$\begin{aligned} p^t_j = \frac{I^t_j}{I^t}, \hspace{0.15cm} \forall j \in SO^t, \forall t \in ST \end{aligned}$$

Evidently, Equation 6 is valid for all periods.

6 $$\begin{aligned} \sum _{j \in SO^t} p^t_j = 1, \hspace{0.15cm} \forall t \in ST \end{aligned}$$

The average probability of selecting hospitals in period *t* is defined as in Equation 7.

7 $$\begin{aligned} {\overline{p}}^{t} = \frac{ \sum _{j \in SO^t} p^t_j}{J}, \hspace{0.15cm} \forall t \in ST \end{aligned}$$

A balance between the probability of selecting hospitals is desired in each period. To measure this, $f_1^{t}$, $\forall t \in ST$ is determined as in Equation 8.

8 $$\begin{aligned} f_1^{t} = \sum _{j \in SO^t} |p^{t}_j-{\overline{p}}^{t}|, \hspace{0.15cm} \forall t \in ST \end{aligned}$$

$t = 1$ represents the current state of the system. Minimizing $f_1^{t}$ for $\forall t > 1$, and satisfying Constraint 9 is expected.

9 $$\begin{aligned} f_1^{t} \le f_1^{1}, \hspace{0.15cm} \forall t > 1 \end{aligned}$$

The balance between the probability of patients choosing hospitals during all periods is measured by $f_1$ defined in Equation 10, which is intended to be minimized. *T* is the number of all periods.

10 $$\begin{aligned} f_1 = \sum _{t \in ST} \frac{f_1^t}{T} \end{aligned}$$

In period *t*, patients’ average time to reach $H_j$ is expressed as in Equation 11.

11 $$\begin{aligned} {\overline{wd}}^t_j = \frac{\sum _{i \in SI^t} wd^t_{ij} x^t_{ij}}{I^t_j}, \hspace{0.15cm} \forall j \in SO^t, \forall t \in ST \end{aligned}$$

In period *t*, the average waiting time before examination in $H_j$ is as in Equation 12.

12 $$\begin{aligned} {\bar{w}}^t_j = \frac{\sum _{i \in SI^t} w^t_{ij} x^t_{ij}}{I^t_j}, \hspace{0.15cm} \forall j \in SO^t, \forall t \in ST \end{aligned}$$

Average total time before examination in $H_j$, including reaching the hospital and waiting time, in period *t* is indicated as in Equation 13.

13 $$\begin{aligned} {\overline{TW}}^t_j = {\overline{wd}}^t_j+{\bar{w}}^t_j, \hspace{0.15cm} \forall j \in SO^t, \forall t \in ST \end{aligned}$$

Average of ${\overline{TW}}^t_j$ for all hospitals is as in Equation 14.

14 $$\begin{aligned} f_2^t = {\overline{TW}}^t = \frac{\sum _{j \in SO^t} ({\overline{wd}}^t_j+{\bar{w}}^t_j)}{J}, \hspace{0.15cm} \forall t \in ST \end{aligned}$$

As defined in Constraint 15, the values of $f_2^t$ in periods should be less than in the current state.

15 $$\begin{aligned} f_2^t \le f_2^1, \hspace{0.15cm} \forall t > 1 \end{aligned}$$

Minimizing $f_2$, defined as in Equation 16, is aimed.

16 $$\begin{aligned} f_2 = \sum _{t \in ST} \frac{f_2^t}{T} \end{aligned}$$

$f_3^t$ is defined as in Equation 17 to measure the balance of ${\overline{TW}}^{t}_j$ between hospitals in period *t*, which is supposed to satisfy Constraint 18.

17 $$\begin{aligned}{} & {} f_3^t = \sum _{j \in SO^t} |({\overline{TW}}^{t}_j)-({\overline{TW}}^{t})|, \hspace{0.15cm} \forall t \in ST \end{aligned}$$

18 $$\begin{aligned}{} & {} f_3^t \le f_3^1, \hspace{0.15cm} \forall t > 1 \end{aligned}$$

The average of $f_3^t$ for all periods is identified as in Equation 19, which is aspired to be minimized.

19 $$\begin{aligned} f_3 = \sum _{t \in ST} \frac{f_3^t}{T} \end{aligned}$$

The total quality acquired by patients choosing $H_j$ in period *t* is defined as in Equation 20.

20 $$\begin{aligned} TQ_j^t = q^t_j I^t_j, \hspace{0.15cm} \forall j \in SO^t, \forall t \in ST \end{aligned}$$

$I^t$, the number of patients in period *t* is calculated as in Equation 21.

21 $$\begin{aligned} I^{t} = \sum _{j \in SO^t} I^t_j, \hspace{0.15cm} \forall t \in ST \end{aligned}$$

In period *t*, the average quality level received by the patients is indicated as in Equation 22.

22 $$\begin{aligned} f_4^t = \frac{TQ^t}{I^t} = \sum _{j \in SO^t} \frac{TQ^t_j}{I^t}, \hspace{0.15cm} \forall t \in ST \end{aligned}$$

The average level of quality over all periods should be at least that of the current situation, which is satisfied by Constraint 23.

23 $$\begin{aligned} f_4^1 \le f_4^t, \hspace{0.15cm} \forall t > 1 \end{aligned}$$

To be maximized, $f_4$ is determined as in Equation 24.

24 $$\begin{aligned} f_4 = \sum _{t \in ST} \frac{f_4^t}{T} \end{aligned}$$

$c_j$ and $c^{ca}_j$ are, respectively, the fixed and unit capacity costs in $H_j$, while $cq_{j}^{t}$ is the cost of the boost in quality in $H_j$ in period *t*; thus, $cq^{t}$ is calculated as in Equation 25.

25 $$\begin{aligned} cq^{t} = \sum _{j \in SO^t} cq_{j}^{t}, \hspace{0.15cm} \forall t \in ST \end{aligned}$$

The total cost of hospitals in period *t* is defined as in Equations 26.

26 $$\begin{aligned} f_5^t = cq^{t} + \sum _{j \in SO^t} (y^t_{j} (c_j+m^t_jc^{ca}_j), \hspace{0.15cm} \forall t \in ST \end{aligned}$$

Due to an increase in capacity, there may be a growth in $f_5^t$. Constraint 27 is defined to manage this augmentation.

27 $$\begin{aligned} f_5^t \le c^t_{max}, \hspace{0.15cm} \forall t \in ST \end{aligned}$$

Previously, we have mentioned that it is assumed that the government manages all hospitals within the addressed system. It is a common governmental objective to upgrade healthcare systems, which can occasionally incur additional costs. Although the resulting enhancements may warrant such expenditures, imposing an upper limit to maintain fiscal responsibility is critical. To this end, Constraint 27 is incorporated into the model to ensure that expenditures do not exceed a limit.

The total cost of hospitals during all periods is determined as in Equations 28, respectively.

28 $$\begin{aligned} f_5 = \sum _{t \in ST} f_5^t \end{aligned}$$

$I^t_{out}$ is the number of patients that the system loses in period *t*; then the probability of patients not choosing the regional hospitals is identified as in Equation 29, which should satisfy Constraint 30.

29 $$\begin{aligned} f^t_6 = \frac{I^t_{out}}{I^t} \end{aligned}$$

30 $$\begin{aligned} f_6^t \le f_6^1, \hspace{0.15cm} \forall t > 1 \end{aligned}$$

The probability that patients do not prefer regional hospitals during all periods is as in Equation 31.

31 $$\begin{aligned} f_6 = \sum _{t \in ST} \frac{f_6^t}{T} \end{aligned}$$

The defined utility for period *t* is an MO function, as in Equation 32.

32 $$\begin{aligned} U^t=(f^t_1, f^t_2, f^t_3, f^t_4, f^t_5, f^t_6) \end{aligned}$$

$f^t_o$, $o=1,2,...,6$ is component *o* of $U^t$. The utility for all periods, i.e., the model’s objective function, is identified as in Equation 33.

33 $$\begin{aligned} U = (f_1, f_2, f_3, f_4, f_5, f_6) \end{aligned}$$

Similarly, $f_o$, $o=1,2,...,6$ is component *o* of *U*. As stated before, it is not intended to minimize $f_5^t$, $\forall t \in ST$, but only to satisfy Constraint 27. This point can be interpreted as the government’s tolerance of cost growth to improve the healthcare system but defining an upper limit. It should be emphasized that due to this reason, the government doesn’t use the Pareto optimality approach for the evaluation of the outputs of the scenarios.

To have a clear understanding of the model, we summarize the assumptions of the model as follows.

- A regional healthcare system is considered, comprising hospitals with varying characteristics. Some hospitals are small and have low capacity and quality, while others are large and have high capacity and quality.
- Despite shorter distances to smaller hospitals, larger hospitals are preferred due to their superior quality.
- Arrival patients to the system choose a hospital considering factors of waiting time and quality.
- The arrival rates of the patients vary in different periods.
- All hospitals in the model are under government administration.
- The government is able to do scenarios like improving hospital capacity and quality as well as periodically closing some hospitals to achieve a more balanced system.
- The government aims to enhance the system based on a defined utility function. The government is willing to accept additional costs to enhance the healthcare system, however, it defines an upper limit for this aim.

## Implementation and experimental results

**Fig. 2:**
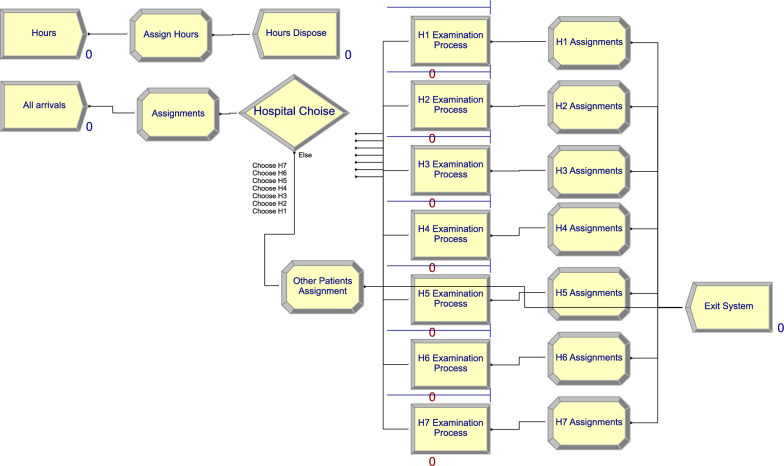
Overview of the Model in Arena Software

The model described in Sect. 3 is implemented in the Rockwell Arena 14 software. We utilize a system with an Intel Core i5 processor, 2.4 GHz, with 12 GB of RAM. Figure 2 presents an overview of the model’s structure as implemented in Arena Software.

In the Create module in the Arena software, the inter-arrival times of patients are modeled using an Exponential distribution. Various components, including the parameter of the Exponential distribution, the decision variable, quality levels, and costs, are identified as variables within the Arena software. Each patient is assigned specific attributes such as quality, waiting time sensitivities, and distances from hospitals. The Set module in the Arena software defines the capacities, i.e., the number of teams available to conduct examinations in hospitals. In the Expression module of the Arena software, Inequality 2 and its associated components are clearly defined. According to this inequality, patients choose a hospital during the simulation. If patient *i* chooses $H_j$ in period *t*, then $x^t_{ij}=1$. This decision is made in the Decide module of the Arena software. Although it is unusual for more than one hospital to fulfill the criteria set by Inequality 2, in cases where this happens, the first hospital that satisfies the inequality is selected. The patients who choose one of the hospitals in the region exit the system after going through a Process module of the Arena software representing the examination process. One of the simulation model’s outputs is the number of patients choosing each hospital, from which the probability of selecting different hospitals is calculated. The other outputs of the simulation model are the waiting times and the utility components for each period.

The details and explanations of the models developed using Arena software are publicly accessible. Links to these resources are provided in the Supporting Information section.

The simulation model is run based on data collected by the authors through a case study conducted in the Eskişehir region of Turkey from 2015 to 2018. Data were collected by observation as well as consulting with experts. Similar to the model of this study, the case study was a regional system that included hospitals that differed in size, capacity, quality of service, and accessibility. This case study aimed to explore strategies for achieving a more balanced healthcare system. More details about the case study can be found in references [16, 17, 37, 38].

There are seven hospitals in the region, indicated as $H_j$, $\forall j=1, ..., 7$. It should be noted that in the case study, $H_1$, $H_2$ and $H_3$ were public hospitals and $H_4$, $H_5$, $H_6$ and $H_7$ private hospitals. But in this study, we assume that all hospitals are public because otherwise, implementing scenarios, especially those about closing hospitals, can be challenging. Although, this assumption does not damage the model’s generalizability, and if multiple hospitals with different characteristics have a unified administration, the proposed model can be applied. It should also be mentioned that, in this study, we consider each hospital as a unit and do not care about the details inside.

Three periods are considered, denoted by $t=1$, $t=2$, and $t=3$. As seen in Table 3, the inter-arrival times of patients are the same in $t=1$ and $t=2$. However, $t=1$ represents the current state, while at $t=2$, the government decides to improve the utility by resectorization. $t=3$ represents the period in which the arrival rates of the patients are less. As seen in the first column of Table 3, based on the arrival rates, the hours of a day are divided into three intervals [16].

**Table 3:** Inter-arrival times, i.e., the average times between patient arrivals (in seconds)

Time intervals	Inter-arrival times	
	*t* = 1 and *t* = 2	*t* = 3
02:00-08:59	65	130
09:00-16:59	30	60
17:00-01:59	19	38

Times to reach $H_1$, $H_2$, and $H_3$ by *i*-th patient in period *t* are considered to be according to the Normal distribution with mean and standard deviation equal to 45 and 15, respectively, while the quality level is equal to 0.9. This is stated as $wd^t_{ij}$ $\sim$ NORM(45,15), $q^t_j=0.9$, $\forall i \in SI^t$, $\forall j=1,2,3$, $\forall t=$1,2,3. Also, we supposed that $wd^t_{ij}$ $\sim$ NORM(20,10), $q^t_j=0.7$, $\forall i \in SI^t$, $\forall j=4,5,6,7$, $\forall t=1,2,3$. This means that the average distance of patients from $H_1$, $H_2$, and $H_3$ is more than $H_4$, $H_5$, $H_6$, and $H_7$, while their quality level is better. The measurement of quality levels was conducted through surveys, the specifics of which are detailed in the study referenced as Teymourifar [37]. In hospitals, $H_1$, $H_2$, and $H_3$, the capacity is supposed to be four, and for hospitals $H_4$, $H_5$, $H_6$ and $H_7$, it is assumed to be two. In this context, “capacity” refers to the number of doctor-nurse teams available to conduct examinations during each shift. $H_1$, $H_2$, and $H_3$ are called large-sized hospitals, and others are called small-sized hospitals. The examination period for each patient is NORM(5,1.5) in all hospitals. If a negative examination time is generated from this distribution, it is assumed to be equal to zero. This means that we overlook the related event, however this issue occurs infrequently. It is also supposed that there is just one examination process. Costs are defined as: $c_j=10000$ and $c^{ca}_j=15000$, $\forall j \in SO^t$. In addition, the cost of a unit increase in the quality level of each hospital is defined as 10000 for all periods. For example, if there is one unit quality improvement in all hospitals in period *t*, then $cq_j^{t}=$10000, and accordingly, using Equation 25, we have $cq^{t}=$70000. The value of $c^t_{max}$ in Constraint 27 is defined as 407,000, which is 10% more than $f_5^1$. Cost values are similar to the years when the case study was conducted, and the unit is Turkish Lira [16]. Furthermore, we suppose that $sn^t_{qu_i}$ $\sim$ NORM(300,10) and $sn^t_{wt_i}=1$. $\forall i \in SI^t$ $\forall t \in ST$. Detailed information regarding the case study, in which the parameter values were determined, is thoroughly documented in Teymourifar [37]. The used parameters are also summarized in Table 4. As it is clear from the values of $y^1_j$, $\forall j \in SO^t$, at $t=1$, all hospitals are available for service, and then $SO^t=SJ$.

The simulation model outputs for the current state are as in Table 4. All results are the average of ten replications, each lasting 720 hours, i.e., one month. As such, each period can also be assumed to be one month. It should be noted that similar outputs are obtained with more repetitions. For example, results similar to those in Table 4 are acquired with 100 replications. The warm-up period is three days. The warm-up period in a simulation helps the model achieve a state where the influences of the initial conditions have faded, ensuring that the data collected afterward reflects the system’s stable status [22].

**Table 4:** Parameters and outputs of the simulation model for the current state of the system, i.e., period $t=1$

Parameters:							
$y^1_1 = y^1_2 = y^1_3 = y^1_4 = y^1_5 = y^1_6 = y^1_7$=1, $q^1_1 = q^1_2 = q^1_3 = 0.9$, $q^1_4 = q^1_5 = q^1_6 = q^1_7 = 0.7$							
$m^1_1 = m^1_2 = m^1_3 = 4$, $m^1_4 = m^1_5 = m^1_6 = m^1_7 = 2$							
Outputs:							
	$H_1$	$H_2$	$H_3$	$H_4$	$H_5$	$H_6$	$H_7$
$I^1_j$	25537	25840	24169	2996	3021	3076	2981
$p^1_j$	0.29	0.29	0.28	0.03	0.03	0.04	0.03
${\bar{w}}^1_j$	22.49	22.15	22.16	0.46	0.46	0.44	0.51
${\overline{wd}}^1_j$	30.23	30.52	30.04	3.67	3.41	3.61	3.39
${\overline{TW}}_j^1$	52.72	52.67	52.20	4.13	3.87	4.05	3.9
$TQ_j^1$	22983.3	23256	21752.1	2097.2	2114.7	2153.2	2086.7
$c_j+m^1_jc^{ca}_j$	70000	70000	70000	40000	40000	40000	40000
$$cq^{1} = 0 , I^1_{out} = 0$$							

Comparing the values of $p^1_j$ and ${\bar{w}}^1_j$, $\forall j \in SO^t$ with the values of the real system in reference [16], the validation of the simulation can be confirmed. It should be noted that the values ${\overline{wd}}^1_j$, $\forall j \in SO^t$ are not given in reference [16], and they are added in this study. In period $t=1$, i.e., the current state, the system is not balanced regarding waiting times and the probability of patients choosing hospitals. Also, $I^1_{out} = 0$, meaning that all patients choose hospitals in the region.

Large-sized hospitals, namely $H_1$, $H_2$, and $H_3$, are more preferred by patients due to their quality. It should be considered that the value of $sn^t_{qu_i}$ is higher than $sn^t_{wt_i}$ in all three periods. It means that patients generally give more importance to quality than waiting time. As a consequence, these hospitals have higher average waiting times than others.

To improve the utility, the following scenarios are designed for period $t=2$:

$$S^2_1$$
: Closing $$H_4$$, $$H_5$$, $$H_6$$, $$H_7$$.
$$S^2_2$$
: Closing $H_4$, $H_5$, $H_6$, $H_7$ and increasing the quality level of $H_1$, $H_2$, $H_3$ to 0.95.
$$S^2_3$$
: Closing $H_4$, $H_5$, $H_6$, $H_7$ and increasing the capacity of $H_1$, $H_2$, $H_3$ to 5.
$$S^2_4$$
: Closing $H_4$, $H_5$, $H_6$, $H_7$ and increasing the capacity of $H_1$, $H_2$, $H_3$ to 5 as well as increasing their quality level to 0.95.
$$S^2_5$$
: Closing $H_4$, $H_5$, $H_6$, $H_7$ and increasing the capacity of $H_1$, $H_2$, $H_3$ to 6.
$$S^2_6$$
: Closing $H_4$, $H_5$, $H_6$, $H_7$ and increasing the capacity of $H_1$, $H_2$, $H_3$ to 6 as well as increasing their quality level to 0.95.
$$S^2_7$$
: Closing $H_4$, $H_5$, $H_6$, $H_7$ and increasing the capacity of $H_1$, $H_2$, $H_3$ to 7.
$$S^2_8$$
: Closing $H_4$, $H_5$, $H_6$, $H_7$ and increasing the capacity of $H_1$, $H_2$, $H_3$ to 7 as well as increasing their quality level to 0.95.
$$S^2_9$$
: Closing $$H_7$$.
$$S^2_{10}$$
: Closing $H_7$ and increasing the quality level of $H_1$, $H_2$, $H_3$ to 0.95.
$$S^2_{11}$$
: Closing $H_7$ and increasing the capacity of $H_1$, $H_2$, $H_3$ to 5.
$$S^2_{12}$$
: Closing $H_7$ and increasing the capacity of $H_1$, $H_2$, $H_3$ to 5 as well as increasing their quality level to 0.95.

Similarly, the following scenarios are designed for period $t=3$:

$$S^3_1$$
: Using the parameters of current state for $t=3$.
$$S^3_2$$
: Closing $$H_4$$, $$H_5$$, $$H_6$$, $$H_7$$.
$$S^3_3$$
: Closing $H_4$, $H_5$, $H_6$, $H_7$ and increasing the quality level of $H_1$, $H_2$, $H_3$ to 0.95.
$$S^3_4$$
: Closing $H_4$, $H_5$, $H_6$, $H_7$ and increasing the capacity of $H_1$, $H_2$, $H_3$ to 5.
$$S^3_5$$
: Closing $H_4$, $H_5$, $H_6$, $H_7$ and increasing the capacity of $H_1$, $H_2$, $H_3$ to 5 as well as increasing their quality level to 0.95.
$$S^3_6$$
: Closing $H_4$, $H_5$, $H_6$, $H_7$ and increasing the capacity of $H_1$, $H_2$, $H_3$ to 6.
$$S^3_7$$
: Closing $H_4$, $H_5$, $H_6$, $H_7$ and increasing the capacity of $H_1$, $H_2$, $H_3$ to 6 as well as increasing their quality level to 0.95.
$$S^3_8$$
: Closing $H_4$, $H_5$, $H_6$, $H_7$ and increasing the capacity of $H_1$, $H_2$, $H_3$ to 7.
$$S^3_9$$
: Closing $H_4$, $H_5$, $H_6$, $H_7$ and increasing the capacity of $H_1$, $H_2$, $H_3$ to 7 as well as increasing their quality level to 0.95.

The parameters of the scenarios designed for periods $t=2, 3$ are summarized in Table 5.

**Table 5:** Parameters in scenarios

$$t=2$$							
$S^2_1$: $y^1_1 = y^1_2 = y^1_3 = 1, y^1_4 = y^1_5 = y^1_6 = y^1_7$=0, $q^1_1 = q^1_2 = q^1_3 = 0.9$, $m^1_1 = m^1_2 = m^1_3 = 4$							
$S^2_2$: $y^1_1 = y^1_2 = y^1_3 = 1, y^1_4 = y^1_5 = y^1_6 = y^1_7$=0, $q^1_1 = q^1_2 = q^1_3 = 0.95$, $m^1_1 = m^1_2 = m^1_3 = 4$							
$S^2_3$: $y^1_1 = y^1_2 = y^1_3 = 1, y^1_4 = y^1_5 = y^1_6 = y^1_7$=0, $q^1_1 = q^1_2 = q^1_3 = 0.9$, $m^1_1 = m^1_2 = m^1_3 = 5$							
$S^2_4$: $y^1_1 = y^1_2 = y^1_3 = 1, y^1_4 = y^1_5 = y^1_6 = y^1_7$=0, $q^1_1 = q^1_2 = q^1_3 = 0.95$, $m^1_1 = m^1_2 = m^1_3 = 5$							
$S^2_5$: $y^1_1 = y^1_2 = y^1_3 = 1, y^1_4 = y^1_5 = y^1_6 = y^1_7$=0, $q^1_1 = q^1_2 = q^1_3 = 0.9$, $m^1_1 = m^1_2 = m^1_3 = 6$							
$S^2_6$: $y^1_1 = y^1_2 = y^1_3 = 1, y^1_4 = y^1_5 = y^1_6 = y^1_7$=0, $q^1_1 = q^1_2 = q^1_3 = 0.95$, $m^1_1 = m^1_2 = m^1_3 = 6$							
$S^2_7$: $y^1_1 = y^1_2 = y^1_3 = 1, y^1_4 = y^1_5 = y^1_6 = y^1_7$=0, $q^1_1 = q^1_2 = q^1_3 = 0.9$, $m^1_1 = m^1_2 = m^1_3 = 7$							
$S^2_8$: $y^1_1 = y^1_2 = y^1_3 = 1, y^1_4 = y^1_5 = y^1_6 = y^1_7$=0, $q^1_1 = q^1_2 = q^1_3 = 0.95$, $m^1_1 = m^1_2 = m^1_3 = 7$							
$S^2_9$: $y^1_1 = y^1_2 = y^1_3 = y^1_4 = y^1_5 = y^1_6 =1, y^1_7$=0, $q^1_1 = q^1_2 = q^1_3 = 0.9$, $q^1_4 = q^1_5 = q^1_6 = 0.7$							
$m^1_1 = m^1_2 = m^1_3 = 4$, $m^1_4 = m^1_5 = m^1_6 = 2$							
$S^2_{10}$: $y^1_1 = y^1_2 = y^1_3 = y^1_4 = y^1_5 = y^1_6 =1, y^1_7$=0, $q^1_1 = q^1_2 = q^1_3 = 0.95$, $q^1_4 = q^1_5 = q^1_6 = 0.7$							
$m^1_1 = m^1_2 = m^1_3 = 4$, $m^1_4 = m^1_5 = m^1_6 = 2$							
$S^2_{11}$: $y^1_1 = y^1_2 = y^1_3 = y^1_4 = y^1_5 = y^1_6 =1, y^1_7$=0, $q^1_1 = q^1_2 = q^1_3 = 0.9$, $q^1_4 = q^1_5 = q^1_6 = 0.7$							
$m^1_1 = m^1_2 = m^1_3 = 5$, $m^1_4 = m^1_5 = m^1_6 = 2$							
$S^2_{11}$: $y^1_1 = y^1_2 = y^1_3 = y^1_4 = y^1_5 = y^1_6 =1, y^1_7$=0, $q^1_1 = q^1_2 = q^1_3 = 0.95$, $q^1_4 = q^1_5 = q^1_6 = 0.7$							
$m^1_1 = m^1_2 = m^1_3 = 5$, $m^1_4 = m^1_5 = m^1_6 = 2$							
$$t=3$$							
$S^3_1$: $y^1_1 = y^1_2 = y^1_3 = y^1_4 = y^1_5 = y^1_6 = y^1_7$=1, $q^1_1 = q^1_2 = q^1_3 = 0.9$, $q^1_4 = q^1_5 = q^1_6 = q^1_7 = 0.7$							
$m^1_1 = m^1_2 = m^1_3 = 4$, $m^1_4 = m^1_5 = m^1_6 = m^1_7 = 2$							
$S^3_2$: $y^1_1 = y^1_2 = y^1_3 =1, y^1_4 = y^1_5 = y^1_6 = y^1_7$=0, $q^1_1 = q^1_2 = q^1_3 = 0.9$, $m^1_1 = m^1_2 = m^1_3 = 4$							
$S^3_3$: $y^1_1 = y^1_2 = y^1_3 =1, y^1_4 = y^1_5 = y^1_6 = y^1_7$=0, $q^1_1 = q^1_2 = q^1_3 = 0.95$, $m^1_1 = m^1_2 = m^1_3 = 4$							
$S^3_4$: $y^1_1 = y^1_2 = y^1_3 =1, y^1_4 = y^1_5 = y^1_6 = y^1_7$=0, $q^1_1 = q^1_2 = q^1_3 = 0.9$, $m^1_1 = m^1_2 = m^1_3 = 5$							
$S^3_4$: $y^1_1 = y^1_2 = y^1_3 =1, y^1_4 = y^1_5 = y^1_6 = y^1_7$=0, $q^1_1 = q^1_2 = q^1_3 = 0.95$, $m^1_1 = m^1_2 = m^1_3 = 5$							
$S^3_6$: $y^1_1 = y^1_2 = y^1_3 =1, y^1_4 = y^1_5 = y^1_6 = y^1_7$=0, $q^1_1 = q^1_2 = q^1_3 = 0.9$, $m^1_1 = m^1_2 = m^1_3 = 6$							
$S^3_7$: $y^1_1 = y^1_2 = y^1_3 =1, y^1_4 = y^1_5 = y^1_6 = y^1_7$=0, $q^1_1 = q^1_2 = q^1_3 = 0.95$, $m^1_1 = m^1_2 = m^1_3 = 6$							
$S^3_8$: $y^1_1 = y^1_2 = y^1_3 =1, y^1_4 = y^1_5 = y^1_6 = y^1_7$=0, $q^1_1 = q^1_2 = q^1_3 = 0.9$, $m^1_1 = m^1_2 = m^1_3 = 7$							
$S^3_8$: $y^1_1 = y^1_2 = y^1_3 =1, y^1_4 = y^1_5 = y^1_6 = y^1_7$=0, $q^1_1 = q^1_2 = q^1_3 = 0.95$, $m^1_1 = m^1_2 = m^1_3 = 7$							

The outlined scenarios primarily focus on strategic adjustments that consider options of closing certain hospitals and enhancing others by increasing their capacity and improving their quality level.

It’s crucial to clarify that the scenarios are not randomly generated but methodically developed based on the opinions of the experts involved in the case study and their viewpoints on strategic healthcare management principles. The scenarios reflect realistic adjustments, mirroring trends and strategies observed in the healthcare system of the case study. This approach ensures that the scenarios represent potential real-world conditions and are grounded in real practices [37]. It is evident that additional scenarios could be incorporated and analyzed, tailored to different systems’ specific needs and capabilities.

The simulation model is run for each scenario. The outputs of the scenarios are presented in Table 6, where the selected ones for each period are bolded. Also, the results from Table 4 are summarized in Table 6.

**Table 6:** The results of scenarios for periods $t=1,2,3$

*t* = 1 (Current state)						
*S*_1_^1^	*f*_1_^1^	*f*_2_^1^	*f*_3_^1^	*f*_4_^1^	*f*_5_^1^	*f*_6_^1^
	0.87	24.79	166.43	0.87	370000	0

*t* = 2						
	*f*_1_2	*f*_2_2	*f*_3_2	*f*_4_2	*f*_5_2	*f*_6_2
$S^2_1$	0.01	22.21	177.68	0.90	210000	0.02
$S^2_2$	0.01	22.21	177.68	0.95	225000	0.02
$S^2_3$	0.00	16.47	131.75	0.90	255000	0.01
$S^2_4$	0.00	16.47	131.75	0.95	270000	0.01
$S^2_5$	0.00	14.13	113.02	0.90	300000	0.00
$S^2_6$	0.00	14.13	113.02	0.95	315000	0.00
$S^2_7$	0.00	13.96	111.69	0.90	345000	0.00
$S^2_8$	**0.00**	**13.96**	**111.69**	**0.95**	**360000**	**0.00**
$S^2_9$	0.79	23.70	169.35	0.88	330000	0.04
$S^2_{10}$	0.79	23.70	169.35	0.93	360000	0.04
$S^2_{11}$	0.99	16.34	130.75	0.90	375000	0.00
$S^2_{12}$	0.99	16.34	130.75	0.95	405000	0.00

*t* = 3						
	*f*_1_3	*f*_2_3	*f*_3_3	*f*_4_3	*f*_5_3	*f*_6_3
$S^3_1$	1.14	13.98	111.86	0.90	370000	0.00
$S^3_2$	0.01	13.97	111.74	0.90	210000	0.00
$S^3_3$	0.01	13.97	111.74	0.95	225000	0.00
$S^3_4$	0.01	13.88	111.02	0.90	255000	0.00
$S^3_5$	**0.01**	**13.88**	**111.02**	**0.95**	**270000**	**0.00**
$S^3_6$	0.01	13.83	110.66	0.90	300000	0.00
$S^3_7$	0.01	13.83	110.66	0.95	315000	0.00
$S^3_8$	0.01	13.82	110.53	0.90	345000	0.00
$S^3_9$	0.01	13.82	110.53	0.95	360000	0.00

For $t=2$, $S^2_8$ is chosen, in which hospitals with less capacity and quality, i.e., $H_4$, $H_5$, $H_6$ and $H_7$ are closed, and the capacity and quality of $H_1$, $H_2$ and $H_3$ are raised. In this case, all the utility components improve, and even the cost is reduced compared to the current state. For $t=3$, $S^3_5$, which is a similar scenario to $S^2_8$, is chosen. However, compared to $S^2_8$, in $S^3_5$, the capacities of $H_1$, $H_2$, and $H_3$ are increased by a smaller amount. The reason is that, at $t=3$, there are fewer arrival rates and, therefore, fewer patients than $t=1, 2$.

It is important to emphasize that the selection of the solution does not adhere to the Pareto optimality approach. As previously discussed, we have set the parameter $c^t_{max}$ in Constraint 27 at 407,000, which represents a 10% increase over the value of $f_5^1$. In $S^2_8$ the value of $f^2_5$ is notably lower than that of $f^1_5$, thereby satisfying the stipulations of Constraint 27. Furthermore, it is evident that in $S^2_8$ the quality metric, $f^2_4$, surpasses that of other solutions, and there is a concurrent enhancement across other objectives when contrasted with both the existing conditions and the scenario $S^2_1$. This signifies that it is feasible to augment objectives, particularly those pertaining to quality, at a cost that is below the one in the current state. While alternative solutions such as $S^2_7$ might also be considered, the salient point is to demonstrate the potential for systemic improvements. Note that the values of $f^2_5$ for both $S^2_7$ and $S^2_8$ are less than $f^1_5$ because the number of hospitals in both is less than that in the current situation. This justification is equally applicable to the choice of $S^3_5$.

Using Equations 10, 16, 19, 24, 28, 31, 33 the results of selected scenarios are summarized in Table 7. These outputs satisfy all Constraints 9, 15, 18, 23, 27 and 30. Also, Table 7 gives the results of using the current state’s parameters for all three periods. As seen, all components of the utility function improve, and even cost reduces.

**Table 7:** The value of the objective functions at the end of the third period, with the proposed scenarios (for three periods)

*f*_1_	*f*_2_	*f*_3_	*f*_4_	*f*_5_	*f*_6_
With proposed scenarios (*S*_8_^2^, *S*_5_^3^ as well as *S*_1_^1^)					
0.29	17.54	129.71	0.92	1000000.00	0.00
With the parameters of current state					
0.96	21.19	148.24	0.88	1110000.00	0.00

### Managerial implications

The study’s findings offer valuable insights for healthcare managers seeking to improve the utility of local healthcare systems. Emphasizing the need for regular resectorization to maintain equilibrium in crucial metrics such as waiting times and demands, these results advocate for a dynamic approach to system management. By adapting proactively to shifting dynamics and aligning resectorization with patient arrival rates, healthcare managers can ensure ongoing system efficiency.

Moreover, the study provides a set of proactive strategies to enhance the overall utility of the system. One such strategy involves the consolidation of healthcare facilities, including the periodic closure of smaller, underutilized hospitals and improvements in the capacity and quality of larger hospitals. Healthcare managers and policymakers can dynamically manage resource allocation and elevate system performance, ultimately improving service quality and patient satisfaction.

A critical managerial insight from this study is that periodically closing some hospitals could lead to a more balanced system. It is important to note that the effectiveness of this strategy depends on how system balance and utility are defined. Nonetheless, we assert that our definition of the system’s utility is consistent with definitions used in the existing literature [42].

The references cited in Section 2 primarily discuss forced closures rather than temporary openings and/or closings. While our study demonstrates that dynamically closing and opening hospitals can benefit the healthcare system, we also anticipate significant repercussions. Policymakers should carefully weigh the pros and cons, considering the financial implications of closing hospitals, reallocating resources, and potentially increasing system volatility. Such drastic changes may introduce stress into the system and adversely impact service quality. Moreover, periodic openings or closings may not seem practical or realistic for some healthcare systems. However, even in such cases, the model can still be used to strategically analyze hospitals’ opening and/or closing from a network design perspective.

Decisions such as closing a hospital are strategic and long-term. Although we use a monthly timeframe in our simulation, this doesn’t necessarily imply that a hospital might close one month and reopen the next. We selected this monthly period to have manageability and computational efficiency in simulation. It’s important to note that this period can vary based on the system’s characteristics. Previous discussions have presented that extending the simulation duration to longer periods does not significantly alter the results, affirming their stability.

For some small-size healthcare systems, it may initially appear intuitive that closing some hospitals could lead to greater balance within the system. However, in this study, we develop a model that allows for a detailed analysis of the impacts of closing specific hospitals, not just in terms of balancing among the remaining ones but also in terms of overall system utility and potential demand loss. It moves beyond intuition by providing quantifiable data and insights, making it a useful tool for decision-making and policy planning in healthcare systems of different sizes.

The study sheds light on a notable challenge, which is the loss of demand for local healthcare systems. An emphasis on community engagement and patient-centered care is instrumental in addressing the challenge of a loss of demand for local healthcare systems. Initiatives that prioritize patient accessibility, satisfaction and convenience can attract and retain patients, ultimately promoting system effectiveness.

Incorporating dynamic decision-making into resource allocation strategies is essential to maintaining efficiency. It ensures that resources are allocated effectively, aligning with the dynamic healthcare landscape. Healthcare administrators should assure a dynamic balance between the outcomes of the system for the benefit of communities.

Administrators should gain insights into the unique needs and barriers of the community, allowing for the development of tailored initiatives. The viability of maintaining smaller, underutilized hospitals should be carefully evaluated. This includes an in-depth analysis of costs and benefits, with the possibility of repurposing such facilities to meet specific healthcare needs or serve as centers for specialized services.

In summary, the proposed approach not only helps to allocate resources but also strengthens the overall utility of the healthcare system, promoting balance.

## Conclusion and future works

This study addresses a regional healthcare system that includes hospitals with dissimilar characteristics in terms of quality, capacity, and average distances to patients. Some hospitals are small with low capacity and low quality, while others are large with higher quality and capacity. Though patients have a shorter average distance to small hospitals, large ones are preferred because of their better quality. We suggest a new model for this system to balance patients’ average waiting times and improve quality. Since balancing is one of the noteworthy objectives of the sectorization concept, the model is characterized based on it. Each hospital and the patients that choose it are considered a sector. The foundation of the proposed model is the definition of a new utility function for the system. Scenarios based on raising the capacity and quality of hospitals to improve utility are designed. In addition, since all of the hospitals in the model are supposed to be managed by the government, closing a subset of them is analyzed in the scenarios. This matter has not been dealt with enough in previous studies. Since the discussed system is dynamic, it is essential to do resectorization over time to improve utility. Therefore, different scenarios should be analyzed over different periods.

We utilize simulation, which is suitable for the analysis of dynamic systems. The model and designed scenarios are implemented within the Rockwell Arena software. In the experimental results section, data from a case study is used. Three periods are defined based on the patients’ arrival rates, one of which is assumed to represent the system’s current state. In the second period, arrival rates are the same as the first one, i.e., the current state, but the government does resectorization to improve the defined utility. In the third period, the arrival rates are lower than the previous ones, for which the government applies resectorization too.

Unlike most of the studies in the literature, we consider the case where patients did not choose any of the regional hospitals. This situation can be regarded as a loss of demand for regional hospitals. In the analysis of scenarios, the cost component in the utility function is handled with a constraint of the upper limit. This means that limited increases in cost can be tolerated if they benefit society. Results demonstrate that the defined scenarios can remarkably improve the utility. The outputs indicate that closing small hospitals and increasing the capacity and quality of large hospitals can improve the defined system. Even in this case, it is possible to reduce the cost. This matter has a noteworthy consequence from a managerial viewpoint. Future research might explore applying our methods to specific healthcare services or specializations rather than hospitals. This matter can be more compatible with recent trends in the field of healthcare management, especially in the post-COVID-19 era, where various countries are implementing policies aiming at a more widespread diffusion of healthcare resources on the territory.

In this work, a high-level and simplified discrete event simulation model is employed to assess a strategic-level problem. Future work will develop more sophisticated techniques to define multiple decision variables. The values of the used parameters, which are selected from a case study [16, 17, 38, 42], affect the managerial implications. In order to provide more broadly applicable managerial consequences, it is intended to analyze a wide variety of parameters in future studies.

Sectorization models are mostly built on integer programming techniques, making finding solutions challenging, especially for large-scale problems [44]. Integer programming models are inherently non-convex due to their constraints’ specific nature, which limit variables to integer values only. This results in a feasible region of separate, isolated points rather than a smooth, continuous area. The non-convex nature of these problems introduces significant challenges in finding optimal solutions, primarily because of the complexity of the solution space and the NP-Hardness of the issues, among other intrinsic difficulties [1, 47]. However, the model described in Sect. 3 diverges from this approach, as it does not employ mathematical programming techniques. It is just a formulation for understanding the problem and implementing it in the Rockwell Arena software. While some studies in the literature, such as Teymourifar [42], employ simulation-based optimization for comparable problems, our approach in this study differs as we utilize simulation solely to assess various scenarios without pursuing optimization.

Assessing the performance of newly developed models and methods presents significant challenges. However, this research underscores the benefits of employing simulation techniques to tackle real-world issues, as the case study exemplifies. Simulation allows for both comprehensible and interpretable outcomes, thus aiding decision-makers and policymakers in making well-informed choices. Furthermore, our study demonstrates the method’s efficiency, with each simulation repetition taking less than one minute to handle scenarios involving more than 85,000 patients, highlighting its feasibility for practical applications.

The model is developed based on the choices of each patient. However, while this approach can be implemented in a simulation model, it can be complicated to define it in a mathematical programming model. Patients may become ill for many reasons that affect their choices, which should be considered in the simulation model. Although the periodic closure of hospitals can facilitate dynamic capacity sharing between healthcare units, in reality, it has managerial complexities. This study implicitly assumes that all hospitals are mutually interchangeable regarding their services. While this assumption may be acceptable in some contexts, it can be overly simplistic and potentially limiting in others, where differences in service quality, specialization, and resource availability between hospitals are significant. Based on the case study [37], we also assume that small hospitals are low-quality, while larger ones are higher quality. If a small hospital can provide higher quality, the results obtained in this study need to be validated. Moreover, we assume shorter distances to smaller hospitals, an assumption that may only hold across some systems.

This study assumes that the government manages all hospitals in the region; otherwise, hospitals’ closure scenarios are not functional. This matter does not affect the model’s generalizability, and it can be applied to hospitals with different characteristics managed by the same authority. Future works will discuss which policies can improve utility where public and private hospitals co-exist in the region. In addition, in this study, we considered just an examination process in the centers. We plan to model complex healthcare systems with diverse processes for future work.

In this research, we focus on the balancing within the healthcare system to enhance its utility. We operate under the premise that all hospitals managed by the government can be strategically opened or closed periodically. This model assumes uniform pricing across these facilities, thus ignoring the role of pricing in hospital choice. The presence of variable pricing under a single government would necessitate another theoretical framework, which is outside the scope of this study. Future research will explore a broader system to accommodate price variations under a single government.

All of the above-mentioned limitations will be considered in future work to develop a more broad-scale model. It’s important to note that the results of this study may not be directly applicable to different inputs; however, the model can be easily adapted to analyze these variations.

## Abbreviations

MO: Multi-objective
NORM: Normal distribution
